# Use of a prospective surveillance model to prevent breast cancer treatment-related lymphedema: a single-center experience

**DOI:** 10.1007/s10549-016-3993-7

**Published:** 2016-09-24

**Authors:** Eun Joo Yang, Soyeon Ahn, Eun-Kyu Kim, Eunyoung Kang, Youngmi Park, Jae-Young Lim, Sung-Won Kim

**Affiliations:** 1 Department of Rehabilitation Medicine, Seoul National University College of Medicine, Seoul National University Bundang Hospital, 300 Gumi-dong Bundang-gu, Seongnam-si, Gyeonggi-do 463-707 Korea; 2 Medical Research Collaborating Center, Seoul National University Bundang Hospital, Seongnam-si, Korea; 3 Department of Surgery, Seoul National University Bundang Hospital, Seoul National University College of Medicine, Seongnam-si, Korea; 4 Daerim St. Mary’s Hospital, Seoul, Korea

**Keywords:** Lymphedema, Surveillance, Breast cancer, Compliance

## Abstract

**Purpose:**

Breast cancer patients undergoing axillary lymph node dissection (ALND) are at risk of lymphedema (LE). Successful management of LE relies on early diagnosis using sensitive modalities. In the current study, we explored the effectiveness of a surveillance program for lymphedema management (SLYM) compared to standard care.

**Methods:**

Breast cancer patients who underwent ALND in Seoul National University Bundang Hospital from January 2008 to December 2015 were included in this prospective study. The SLYM commenced in May 2011. The LE outcomes of patients treated prior to initiation of the SLYM were compared with those of patients after SLYM implementation.

**Results:**

A total of 707 patients were included, 390 in the SLYM group and 317 in the historical control (HC) group. A total of 203 patients (28.7 %) had episodes of all-stage LE during follow-up. Of these, 126 (19.7 %) were in the surveillance group and 77 (24.3 %) in the HC group. The overall 5-year cumulative incidence of LE (greater than stage 3) was 25 (95 % CI 15.4–34.6) (6.4 %) in the SLYM group and 48 (95 % CI, 15.4–34.6) (15.1 %) in the HC group. In the SLYM group, poor compliance had a significant impact on LE incidence (*OR* = 2.98, *P* = 0.002). Low level of self-monitoring and insight scores were significantly related to LE incidence (*OR* = 1.31, *P* = 0.025) after adjusting for age, body mass index, the type of surgery chosen, radiation therapy, and chemotherapy. With a cut-off of 29.5 days from operation to the first visit to the LE clinic, the sensitivity was 60 % and the specificity 61 % in terms of predicting a LE event.

**Conclusions:**

Surveillance improves LE prevention compared to clinical evaluation. The first visit to the LE clinic should be made within 1 month after surgery. In the first year, visits should be made at intervals of less than 3 months.

**Electronic supplementary material:**

The online version of this article (doi:10.1007/s10549-016-3993-7) contains supplementary material, which is available to authorized users.

## Introduction

Breast cancer survivors are at increased risk for the development of breast cancer-related lymphedema (BCRL), a chronic, debilitating, and disfiguring condition that is progressive and requires lifelong self-management of symptoms. Lymphedema (LE) is caused by a disruption of the lymphatic system that, in the initial stages, leads to fluid accumulation in the interstitial tissue spaces, and eventually manifests clinically as swelling of the arm, breast, shoulder, neck, or torso [1]. Early assessment and intervention may be important to correct subtle subclinical LE that, if left untreated, may progress to chronic and severe LE. Previous studies suggested that regular surveillance of upper-body morbidities such as LE should be integrated into the routine postoperative care of women with breast cancer, as early diagnosis potentially contributes to more effective management, and prevention of progression of troublesome conditions [2].

Detection and management of early-stage LE may prevent progression to chronic disabling disease [3] and may enable cost-effective conservative intervention. Fu et al. [4] found that patient education on the early signs and symptoms of upper-body morbidity, in particular disease progression, was important. Bioimpedance spectroscopy (BIS) assesses changes in extracellular fluid levels and can identify such changes in limbs prior to clinical presentation (thus before the condition becomes non-pitting [fibrotic] [5]). A short trial showed that compression garments effectively treated subclinical LE [6]. A systematic review revealed that compression garments and bandages reduced the volume of cancer-related LE.

A prospective surveillance model may be useful to detect BCRL at an early stage, when the opportunities to reduce risk or slow progression are optimal. A surveillance program would allow healthcare providers to detect BCRL symptoms early, affording better opportunities to prevent progression to the subclinical stage and to institute continuous care plans from the inpatient to the outpatient settings [6]. Few rigorous comparative research studies have been performed on patients with BCRL, compromising the development of evidence-based assessment of, and treatments for, hundreds of thousands of women who have, or are at risk for the development of, BCRL. Therefore, we hypothesized that a surveillance program featuring the use of extracellular water (ECW) ratio to detect subclinical LE might be effective to prevent the development of LE of stages 2 and 3 after surgery.

The purpose of our study was thus to evaluate the efficacy of a surveillance program including ECW ratio measurement in terms of detection of subclinical LE; we explored whether such detection might prevent the development of advanced LE (stages 2 and 3) after surgery.

## Patients and methods

### Study design

We accessed the database of the Seoul National University Bundang Hospital and extracted records made from January 1, 2008, through to December 31, 2015. We collected data on women aged 19–99 years who were newly diagnosed with stage 1–3 unilateral breast cancer and who had undergone breast cancer surgery with axillary lymph node dissection (ALND). This was thus a single-center observational study. Clinical characteristics, demographic information, treatment details, and LE status upon follow-up were all recorded in our clinical database warehouse system. A surveillance program for LE management (SLYM) commenced in May 2011. We sought to identify patients with early-stage LE and institute management at that time.

The 767 patients were divided into three groups: one historical comparison (HC) group treated prior to implementation of the surveillance program (thus, from 2008 through 2010; HC group, *n* = 317); those who were screened from 2010 to 2011 (group B, *n* = 60); and a current group (treated from 2011 through 2015) who were participating in the surveillance program (SLYM group, *n* = 390).

The primary aim of our study was to compare the event-free survival rate between the HC and SLYM groups. This was defined as survival without advanced LE. LE was defined by reference to the guidelines of the International Society of Lymphology (ISL) consensus document. These guidelines feature a staging system based on the amount of swelling and the condition of the skin and tissues, and can be used to identify disease progression and severity and the potential for successful treatment. We defined the event of interest as advanced LE (greater than stage 3). The secondary aim was to measure the incidence of LE of any stage during follow-up.

Three physicians defined LE stages by reference to the ISL criteria. Inter-rater agreement between physicians A (a psychiatrist, EJY), B (a surgeon, SWK), and C (a surgeon, EYK) was analyzed by calculation of kappa coefficients. We used simple randomization to select representative samples from the pre-surveillance (May 2010 to April 2011) (*n* = 45) and post-surveillance (May 2012 to April 2013) (*n* = 50) periods. Kappa coefficients were 0.87 and 0.93 in the pre- and post-surveillance period, respectively. ICCs were 0.84 and 0.92 in the pre- and post-surveillance period, respectively. Overall agreements (%) were 81.2 and 83.7 % in the pre- and post-surveillance period, respectively.

### Surveillance protocol

The SLYM program was implemented in May 2011 to identify high-risk LE patients who would benefit from comprehensive surveillance by a transdisciplinary team, with an emphasis on early detection and prevention of LE. A care plan was initiated immediately after surgery for all patients who underwent ALND to identify patients at high risk of LE.

To detect subclinical LE, we used a reliable and valid instrument, the breast cancer and lymphedema symptom experience index [7, 8] and a multi-frequency bioelectrical impedance analyzer (BIA) (Inbody S10 Biospace, Biospace Co. Ltd., Korea; Model JMW140), according to the manufacturer’s instructions. The BIA estimates bodily composition by comparing conductivity differences between various tissues; these reflect the biological characteristics of the tissues. Electrodes are placed at eight precisely defined tactile points prior to multi-segmental frequency analysis. A total of 30 impedance measurements are obtained at six different frequencies (1, 5, 50, 250, 500, and 1000 kHz) from the following five locations: the right and left arms, the trunk, and the right and left legs. Each single-frequency bioimpedance ratio is expressed as a Z_unaffected limb_/Z_affected limb_ ratio, yielding LE index values >1 [9].

The inclusion criterion for compressive intervention was a diagnosis of subclinical LE. The diagnostic criteria included subjective LE symptoms and an ECF ratio >1.066 at the dominant arm, or a ratio >1.106 at the non-dominant arm. The garment provided was a 20–30 mm-Hg compression sleeve (with a gauntlet) fitted by a trained nurse. If the volume increased by >3 %, both a compression garment and education on manual lymphatic massage were prescribed for 4 weeks.

If the clinical LE stage is greater than two, complete decongestive therapy (CDT) performed by specially trained LE therapists, is considered to be the international standard of care [10, 11]. Such therapy seeks to move lymphatic fluid to an area from which it may drain, thus reducing swelling. The therapy features manual lymph drainage, progressive active and action-assisted exercises, and compression therapy [12].

If LE recurred, we checked the status thereof using the same tools. Patients with stage 0 or 1 subclinical LE commenced progressive strengthening exercises; they were instructed in modified progressive weight-lifting exercises [13]. Patients participated in a supervised program run in the hospital and continued the program at home. Strengthening exercises commenced with the aid of a thera-band. After checking shoulder strength using a manual muscle strength test, an appropriate thera-band was prescribed, as was the number of exercise repetitions. This number was progressively increased if no symptoms were evident after three sessions. If the fluid volume decreased after intensive treatment, the patients returned to 3-month surveillance until 1 year after surgery, and were then screened at 6-month intervals.

Compliance with the surveillance program was categorized as good or poor by reference to the intervals between visits to the LE clinic. The times from the day of operation to the first visit to the LE clinic, and those between follow-up days, were calculated. The cut-off intervals were 3 months (good compliance ≤ 3 months; poor compliance >3 months).

To assess health-related empowerment, patients were asked to complete the Health Education Impact Questionnaire (HeiQ) at their first visit. The HeiQ is a well-validated, widely used instrument developed in Australia, containing 40 questions exploring eight different domains (health-directed behavior, positive and active life engagement, emotional well-being, self-monitoring and insight, constructive attitudes and approaches, skill and technique acquisition, social integration and support, and health service navigation) [14]. We analyzed the self-monitoring and insight dimension scores (ranges 1.0–4.0) and categorized them into low (1.0–2.0) and high level (3.0–4.0).

### Historical control group (HC group)

In the HC group, LE was diagnosed when patients complained of subjective symptoms, or by clinicians of our multidisciplinary team. Patients who complained and those with swelling on the operative site (noted by clinicians) were referred to the LE clinic for further evaluation and management. If LE was confirmed, CDT was applied.

### Statistical considerations

The actuarial rates of irreversible LE were calculated using the Kaplan–Meier method. All statistics were calculated from the date of surgery. To isolate the effect of the SLYM program, our calculation of the high risk-free survival rate in the SLYM group included only survival without advanced (over stage 3) LE. Cox’s proportional hazard model was used to compare the high risk-free survival rates between groups after adjusting for age, body mass index, the number of cycles of chemotherapy given, and the radiotherapy field. Only variables that were significant upon univariate analysis were included in multivariate analysis. Comparisons between groups were performed with the aid of the log-rank test. *P* values <0.05 were deemed to be statistically significant. All statistical tests were two-sided and were performed with the aid of SPSS (version 17.0) software (SPSS Inc., Chicago, IL).

## Results

### Patient characteristics

Patient and treatment characteristics are summarized in Table 1. The median age at the time of surgery was 47.9 years (range, 23–89 years). Breast-conserving surgery (BCS) was performed on 138 (35.4 %) and mastectomy on 252 (64.6 %) patients in the SLYM group, and 28 (8.8 %) and 289 (91.9 %) patients in the HC group. Chemotherapy (doxetaxel) was administered to 280 patients (71.9 %) in the SLYM group and 223 (70.3 %) in the HC group. Radiotherapy was prescribed for 351 patients (90.0 %) in the SLYM group, of whom 211 (54.0 %) received breast or chest-wall irradiation alone, and for 276 in the HC group (87.1 %), of whom 174 (54.9 %) received breast or chest-wall irradiation alone.

**Table 1:** Patient and treatment characteristics

	HC group (*N* = 317), *n* (%)	SLYM group, (*N* = 390), *n* (%)
Age at diagnosis (years)	48.6 ± 11.7 (25–82)	47.6 ± 10.7 (23–89)
BMI	23.3 ± 9.8	24.3 ± 9.5
Dominant side	132 (41.6 %)	207 (53.0 %)
Histopathologic stage		
I(%)	92 (29.0 %)	109 (28.0 %)
II (%)	168 (53.0 %)	215 (55.0 %)
III (%)	57 (18.0 %)	66 (17.0 %)
Breast surgery		
BCS	28 (8.8 %)	138 (35.4 %)
Mastectomy	289 (91.1 %)	252 (64.6 %)
Radiotherapy		
Not done	41 (12.9 %)	47 (12.0 %)
Breast only	174 (54.9 %)	211 (54.0 %)
Breast and SCRT (%)	102 (32.2 %)	140 (36.0 %)
Chemotherapy		
Doxetaxel	223 (70.3 %)	280 (71.9 %)
Cycle of doxetaxel	5.1 ± 0.3	6.1 ± 0.2

*HC* historical control, *SLYM* surveillance program for lymphedema management, *BCS* breast-conserving surgery, *SCRT* supraclavicular radiation therapy

### Incidence and time course of LE

Initially, 203 patients (28.7 %) were found to have developed LE of any stage during follow-up. Of these, 126 (19.7 %) were in the SLYM group and 77 (24.3 %) in the HC group. Of these patients, 131 (18.5 %) in whom LE had resolved or improved at the next follow-up (thus after 6 months) were defined as having reversible LE. Of these patients, 101 (33.6 %) were in the SLYM group and 30 (9.5 %) in the HC group. The initial stage of the first swelling episode was two in 179 patients (25.3 % of all patients; 116 in the SLYM group and 63 in the HC group) and three in 24 patients (3.4 %) (Ten in the SLYM group and 14 in the HC group). A total of 48 (6.8 %) patients who were initially of stage 2 progressed to stage 3 (15 in the SLYM group and 33 in the HC group).

**Fig. 1:**
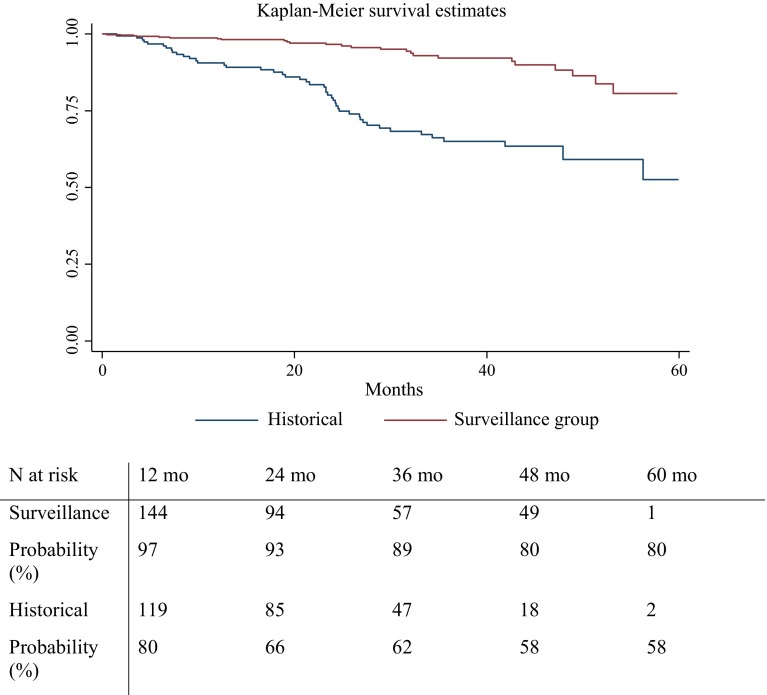
Kaplan–Meier with log-rank analysis for the irreversible lymphedema in the surveillance group compared the historical control group

The overall 5-year cumulative incidence of advanced LE (greater than stage 3) was 25 (95 % CI 15.4–34.6 (6.4 %) in the SLYM group and 48 (95 % CI 15.4–34.6) (15.1 %) in the HC group (Fig. 1). The median interval from surgery to initial swelling in patients with advanced LE was 25.4 months (95 % CI 18.6–32.1) in the SLYM group and 20.7 months (17.0–24.5 months) in the HC group.

The overall 5-year cumulative incidence of LE (any stages) was 126 (95 % CI 106.8–145.2 (32.3 %) in the SLYM group and 145 (95 % CI 127.6–162.4) (45.7 %) in the HC group. The median interval from surgery to initial swelling in patients with any stage of LE was 16.9 months in the SLYM group and 18.4 months in the HC group. Of the 77 affected patients, LE first occurred within 1 year of diagnosis in 97 and 80 % of patients, and within 3 years in 89 and 62 %, in the SLYM and HC group, respectively.

### Risk factors for LE

Upon multivariate analysis, the following treatment-related factors were significantly correlated with an increased risk of LE: chemotherapy with docetaxel (hazard ratio (HR) 4.98; *P* = 0.001) and radiotherapy on breast with SCRT (HR 1.20; *P* = 0.045) (Table 2). The HR of the pathological stage was 1.52, and lacked significance.

**Table 2:** Multivariate analysis of risk factors associated with lymphedema (*N* = 707)

Clinical characteristics	HR	95 % CI	*P* value^a^
Age (≥60 years)	0.03	0.01–0.05	0.014
BMI (≥25 kg/m^2^)	1.60	0.69–2.75	0.255
Dominant side	1.75	0.45–8.63	0.432
Histopathologic stage (≥II)	1.52	0.73–3.11	0.321
Type of surgery (mastectomy)	1.17	0.42–3.29	0.766
Radiation therapy (breast with SCRT)	2.01	1.05–3.03	0.045
Chemotherapy (taxel)	4.98	1.93–12.87	0.001
Surveillance protocol	0.31	0.17–0.56	<0.001

*HR* hazard ratio, *CI* confidence interval, *SCRT* supraclavicular radiation therapy

^a^ Cox proportional hazards model

### Risk factors for LE in the SLYM group

Table 3 shows the results of multivariate logistic regression modeling. Model 1 computed coefficients for a LE event using demographic characteristics such as age, body mass index (BMI), and the dominant side. None of age, BMI, or the operative site was significantly associated with a LE event.

**Table 3:** Multiple logistic regression analysis of patients with breast cancer in surveillance group (*N* = 390)

Variables	Model 1	Model 2	Model 3
Age	1.01 (0.98–1.05)	1.01 (0.98–1.05)	1.02 (0.98–1.05)
BMI	1.02 (0.97–1.06)	1.02 (0.97–1.05)	1.03 (0.98–1.05)
Dominant side	1.05 (0.96–1.16)	1.06 (0.96–1.16)	1.06 (0.97–1.15)
Type of surgery (mastectomy)		1.70 (0.86–6.55)	1.67 (0.88–6.11)
Radiation therapy (breast with SCRT)		2.02 (1.06–3.11)^*^	2.01 (1.05–3.10)^*^
Chemotherapy (taxel)		5.56 (2.00–9.11)^*^	5.55 (1.99–9.01)^*^
Poor compliance (interval of follow-up >3 months)			3.16 (1.36–6.89)^*^
Low grade of self-monitoring and insight (score ≤2)			1.31 (1.03–3.24)^*^

Values are odds ratio (95 % CI)

Model 1: Age, BMI, and dominant side

Model 2: Model 1 + histopathologic stage, type of surgery, radiation therapy, chemotherapy

Model 3: Model 2 + compliance, self-monitoring, and insight

*SCRT* supraclavicular radiation therapy

* *P* < 0.05

Model 2 incorporated cancer and treatment characteristics, such as histopathological stage, type of breast surgery, radiotherapy, and chemotherapy (docetaxel), in addition to demographic characteristics. Chemotherapy and radiotherapy on breast with SCRT were significantly related to the development of LE events (*OR* = 5.56, *P* = 0.032; *OR* = 2.02, *P* = 0.243, respectively).

**Fig. 2:**
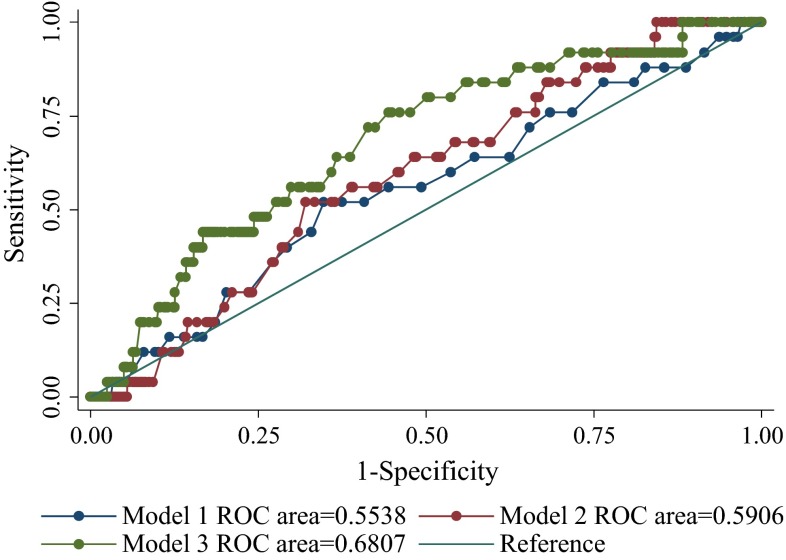
ROC curve of lymphedema prediction in surveillance group

Model 3 incorporated compliance with the surveillance program in addition to demographic and treatment characteristics. Chemotherapy and radiation therapy remained significantly associated with LE events (*OR* = 5.55, *P* = 0.041; *OR* = 2.01, *P* = 0.042, respectively). Poor compliance had a significant impact on LE events (*OR* = 2.98, *P* = 0.002). The low level of self-monitoring and insight were significantly related to LE events (*OR* = 1.31, *P* = 0.025) (Fig. 2).

We explored the sensitivity and specificity of a cut-off value (days) from operation to the first visit to the LE clinic in terms of predicting a LE event. With a cut-off of 29.5 days, the sensitivity was 60 % and the specificity 61 %. However, the area under the ROC curve was 0.6417, thus affording poor diagnostic utility. However, 29.5 days can be used as a reference value (Supplementary Table).

## Discussion

The frequency of advanced LE after breast cancer surgery was reduced on the introduction of a LE surveillance program. The program afforded ten percentage points of the total reduction in advanced LE among women invited to participate. The first visit to the LE clinic should be made within 1 month after surgery. In the first year, visits should be made at intervals of less than 3 months.

Early LE detection is important, and can prevent development of irreversible disease. Surveillance can prevent progression of subclinical LE [6]. No evidence-based method for early detection of subclinical LE is yet available. Relevant clinical trials, using various surveillance protocols, are in progress [15]. However, few rigorous comparative studies on patients with BCRL have appeared, compromising the development of evidence-based assessment and treatment for women who have LE or are at risk of LE development [15]. We found that the hazard ratio for LE development was three times higher in the control than the surveillance group.

We used BIS to measure extracellular fluid volumes; BIS records the responses to an applied electrical current, and is more sensitive and specific than conventional methods [16]. BIS allows earlier recognition of subclinical LE, before the disease is clinically evident upon tape measurement of water displacement. Soran et al. [17] reported that periodic monitoring of women at high risk of LE, using BIS, facilitated early detection and timely intervention.

Currently, the lack of data on appropriate assessment frequency and the assessor skills required compromise analyses of the cost-effectiveness of prospective surveillance models for breast cancer survivors [18]. One previous study [17] recorded measurements preoperatively, at 3–6 months after surgery, and then annually for 5 years. Other studies assessed patients five times: preoperatively and every 3 months for the following year [6, 19]. Clearly, such variations affect costs. The incremental benefits afforded by surveillance at 3- versus 4- or 6-month intervals warrants study [18]. We explored the sensitivity and specificity of time from surgery to the onset of surveillance in terms of predicting a LE event. Surveillance should commence within 1 month of surgery; this should be considered when planning clinical interventions and the follow-up schedule. A previous study also suggested that the first postoperative assessment visit should take place within 1 month after surgery [20].

Of the patient-, treatment-, and disease-related factors, chemotherapy with doxetaxel was associated with an increased risk of LE. Neither age nor BMI correlated with development of a LE event. An earlier study found that these factors were, in fact, important [21], but another study found no such correlations [22]. Radiation therapy of the breast, and SCRT, were significantly associated with LE development. Regional irradiation is considered to be a significant risk factor for LE [23]. Irradiation of the breast and SCRT increased the risk of LE compared to that associated with breast irradiation alone [24]. The question of whether chemotherapy is a risk factor for the development of lymphedema remains controversial. Patients receiving docetaxel-based chemotherapy were at an increased risk of developing LE [21].

Adherence to self-management regimens must be improved. Adherence to the surveillance program was checked by recording the intervals between visits to the in-hospital program. The self-monitoring and insight scales capture the ability of an individual to monitor a medical condition, triggering physical responses that create insight and appropriate self-management [25]. We found that adherence and self-monitoring were significantly associated with LE development. Visits should be at less than 5-month intervals. After adjusting for cancer and treatment factors, compliance with the surveillance program remained significant. Cancer patients require health-related empowerment to manage the challenges of cancer and to control their lives [26, 27]. Especially in patients at high risk of LE, fear or denial operates against disease prevention and management. All surveillance programs must seek to empower patients; this is becoming increasingly important [28].

Caution must be exercised before seeking to generalize our results. Although our choice of a matched cohort may minimize the risk of systematically overestimating the effects of the surveillance program, the nonrandomized design of our work is a major limitation. Is it possible that the lead time created bias when the frequencies of incident-based LE were calculated? We counted LE events developing after breast cancer surgery only if the LE was diagnosed within a group. For example, in the surveillance group, advanced LE was attributed to breast cancer only if the disease was diagnosed early by means of surveillance or was clinically diagnosed while the woman in question was in fact in the group. However, for women diagnosed early during surveillance, the initial clinical diagnosis would have been made at an unknown time within the study period. Thus, the lead time played no role when we calculated LE rates. We believe, therefore, that the calculations for the two groups are free of such bias.

We evaluated the effects of a surveillance program compared to self-reporting/clinical evaluation. Our results provide a rationale for the future randomized clinical trials required to validate our program in patients at high risk of LE. We conclude that surveillance reduces the rate of LE developing after surgery. The magnitude of the benefit was modest. Most importantly, the apparent benefit afforded by optimized patient care may be lost unless patients are empowered in terms of self-assessment and self-monitoring. The take-home message is that the LE surveillance program achieved an absolute reduction of ten percentage points in the frequency of advanced LE developing after breast cancer surgery.

## Electronic supplementary material

Below is the link to the electronic supplementary material.

Supplementary material 1 (DOCX 19 kb)
