# Supplementary material for: Use of a prospective surveillance model to prevent breast cancer treatment-related lymphedema: a single-center experience
Source: Breast Cancer Res Treat. 2016 Sep 24;160(2):269–76. doi: 10.1007/s10549-016-3993-7 (PMC5065580; doi:10.1007/s10549-016-3993-7)
Supplement: Supplementary file 1 — Supplementary material 1 (DOCX 19 kb) [file 10549_2016_3993_MOESM1_ESM.docx]

Supplemental table

| Cut-off value (day) | sensitivities | specificities |
| --- | --- | --- |
| 1.5 | 1 | 0.00 |
| 4.0 | 1 | 0.02 |
| 5.5 | 1 | 0.03 |
| 6.5 | 1 | 0.04 |
| 7.5 | 1 | 0.05 |
| 8.5 | 1 | 0.08 |
| 9.5 | 1 | 0.09 |
| 10.5 | 0.96 | 0.13 |
| 11.5 | 0.92 | 0.20 |
| 12.5 | 0.92 | 0.22 |
| 13.5 | 0.8 | 0.33 |
| 14.5 | 0.8 | 0.36 |
| 15.5 | 0.8 | 0.38 |
| 16.5 | 0.8 | 0.40 |
| 17.5 | 0.8 | 0.43 |
| 18.5 | 0.8 | 0.47 |
| 19.5 | 0.8 | 0.47 |
| 20.5 | 0.72 | 0.51 |
| 21.5 | 0.68 | 0.52 |
| 22.5 | 0.68 | 0.53 |
| 23.5 | 0.68 | 0.54 |
| 24.5 | 0.68 | 0.55 |
| 25.5 | 0.64 | 0.57 |
| 26.5 | 0.64 | 0.57 |
| 27.5 | 0.6 | 0.59 |
| 28.5 | 0.6 | 0.60 |
| **29.5** | **0.6** | **0.61** |
| 31.0 | 0.56 | 0.62 |
| 32.5 | 0.56 | 0.62 |
| 33.5 | 0.56 | 0.62 |
| 34.5 | 0.56 | 0.63 |
| 36.0 | 0.52 | 0.65 |
| 37.5 | 0.52 | 0.66 |
| 38.5 | 0.52 | 0.66 |
| 40.0 | 0.52 | 0.67 |
| 41.5 | 0.52 | 0.68 |
| 42.5 | 0.52 | 0.69 |
| 43.5 | 0.52 | 0.70 |
| 45.0 | 0.52 | 0.70 |
| 46.5 | 0.52 | 0.71 |
| 47.5 | 0.52 | 0.71 |
| 48.5 | 0.52 | 0.73 |
| 49.5 | 0.52 | 0.74 |
| 50.5 | 0.52 | 0.74 |
| 52.0 | 0.52 | 0.75 |
| 54.5 | 0.48 | 0.75 |
| 56.5 | 0.44 | 0.75 |
| 58.0 | 0.44 | 0.75 |
| 60.5 | 0.44 | 0.75 |
| 63.0 | 0.44 | 0.76 |
| 64.5 | 0.44 | 0.76 |
| 66.0 | 0.44 | 0.76 |
| 68.5 | 0.44 | 0.77 |
| 71.5 | 0.44 | 0.77 |
| 75.0 | 0.44 | 0.77 |
| 81.0 | 0.44 | 0.77 |
| 86.0 | 0.44 | 0.78 |
| 90.0 | 0.44 | 0.78 |
| 93.5 | 0.44 | 0.78 |
| 94.5 | 0.44 | 0.79 |
| 96.5 | 0.44 | 0.79 |
| 99.0 | 0.44 | 0.79 |
| 104.0 | 0.44 | 0.79 |
| 109.5 | 0.4 | 0.79 |
| 111.5 | 0.4 | 0.80 |
| 113.0 | 0.4 | 0.80 |
| 116.5 | 0.4 | 0.81 |
| 121.0 | 0.4 | 0.81 |
| 126.5 | 0.4 | 0.81 |
| 130.5 | 0.36 | 0.81 |
| 132.0 | 0.36 | 0.82 |
| 134.0 | 0.36 | 0.82 |
| 136.0 | 0.36 | 0.82 |
| 140.0 | 0.36 | 0.83 |
| 144.0 | 0.36 | 0.83 |
| 150.5 | 0.36 | 0.83 |
| 162.5 | 0.32 | 0.83 |
| 172.0 | 0.32 | 0.84 |
| 176.0 | 0.32 | 0.84 |
| 179.0 | 0.32 | 0.84 |
| 181.5 | 0.32 | 0.84 |
| 183.0 | 0.28 | 0.84 |
| 184.5 | 0.28 | 0.85 |
| 186.5 | 0.24 | 0.85 |
| 188.5 | 0.24 | 0.85 |
| 189.5 | 0.24 | 0.85 |
| 190.5 | 0.24 | 0.86 |
| 191.5 | 0.24 | 0.86 |
| 193.5 | 0.24 | 0.86 |
| 195.5 | 0.24 | 0.87 |
| 197.0 | 0.24 | 0.87 |
| 201.0 | 0.16 | 0.87 |
| 205.0 | 0.16 | 0.88 |
| 206.5 | 0.16 | 0.88 |
| 210.0 | 0.16 | 0.88 |
| 213.5 | 0.12 | 0.88 |
| 215.5 | 0.12 | 0.89 |
| 218.5 | 0.12 | 0.89 |
| 223.0 | 0.12 | 0.89 |
| 226.5 | 0.12 | 0.90 |
| 233.5 | 0.12 | 0.90 |
| 246.0 | 0.12 | 0.90 |
| 253.5 | 0.12 | 0.90 |
| 258.5 | 0.12 | 0.91 |
| 267.0 | 0.12 | 0.91 |
| 272.5 | 0.12 | 0.91 |
| 274.0 | 0.12 | 0.92 |
| 277.0 | 0.12 | 0.92 |
| 280.5 | 0.08 | 0.92 |
| 287.5 | 0.08 | 0.92 |
| 300.5 | 0.08 | 0.92 |
| 316.5 | 0.04 | 0.92 |
| 330.0 | 0.04 | 0.93 |
| 346.0 | 0.04 | 0.93 |
| 365.0 | 0.04 | 0.93 |
| 379.5 | 0.04 | 0.94 |
| 390.0 | 0.04 | 0.94 |
| 398.0 | 0.04 | 0.95 |
| 409.5 | 0.04 | 0.95 |
| 423.0 | 0.04 | 0.95 |
| 435.5 | 0.04 | 0.95 |
| 455.0 | 0.04 | 0.96 |
| 493.0 | 0.04 | 0.96 |
| 520.0 | 0.04 | 0.96 |
| 525.5 | 0.04 | 0.97 |
| 561.5 | 0 | 0.97 |
| 602.0 | 0 | 0.97 |
| 621.0 | 0 | 0.97 |
| 666.0 | 0 | 0.97 |
| 756.0 | 0 | 0.98 |
| 897.5 | 0 | 0.98 |
| 1019.5 | 0 | 0.99 |
| 1085.0 | 0 | 0.99 |
| 1144.0 | 0 | 0.99 |
| 1180.0 | 0 | 0.99 |
| 1240.5 | 0 | 1.00 |
